# Prevalence and Associated Factors of Cyberchondria: A Scoping Review

**DOI:** 10.1155/tswj/9950027

**Published:** 2026-01-22

**Authors:** Daniel Miezah, Rejoice Adzakpa, Emmanuella Mawuena Ama Bani, Paul Obeng

**Affiliations:** ^1^ Department of Education and Psychology, University of Cape Coast, Cape Coast, Ghana, ucc.edu.gh; ^2^ Department of Health, Physical Education and Recreation, University of Cape Coast, Cape Coast, Ghana, ucc.edu.gh

## Abstract

Cyberchondria, defined as heightened health anxiety and distress arising from excessive online searches about medical symptoms or risks, is an emerging mental health concern in the digital era. However, less synthesized evidence exists on its prevalence, associated factors, and their impact on health. This scoping review synthesized evidence on its prevalence, associated factors, and impacts. Following Arksey and O′Malley′s framework, four databases (PubMed, Scopus, JSTOR, Dimensions), alongside Google Scholar and reference lists, were systematically searched. A total of 42 studies were included. Prevalence estimates ranged from 30.7% to 55.6%, with consistent links to health anxiety, internet addiction, and anxiety sensitivity. Demographic variations were observed by age, gender, and occupation. Significant predictors included health anxiety, depression, stress, and maladaptive metacognitive beliefs. Reported consequences included self‐diagnosis, self‐medication, and reliance on safety behaviors highlighting risks for inappropriate healthcare use and adverse mental health outcomes. Addressing these findings requires targeted interventions that promote digital health literacy, responsible online health information‐seeking, and early identification of individuals at risk of excessive health‐related internet use. Future longitudinal and cross‐cultural research should build on these correlates and predictors to clarify causal pathways and inform evidence‐based prevention and treatment strategies.

## 1. Introduction

A significant portion of the global population currently depends on the Internet for their daily activities. In 2021, approximately 4.9 billion people, constituting 63% of the world′s population, were using the Internet. This shows a 17% increase compared with 2019, with approximately 782 million people accessing the Internet [1]. Besides, the Internet has become a primary source of health‐related information for many individuals, with studies indicating that up to 72% of internet users have searched for health‐related content online [2]. However, an estimated 40% of health‐related websites lack rigorous, research‐based content, with information that may be unclear, unreliable, exaggerated, or false [3]. For individuals with limited medical knowledge, reliance on the Internet for self‐diagnosis can significantly heighten anxiety, as exposure to unverified or alarmist information may lead to unnecessary worry or misinterpretation of symptoms, making cyberchondria a public health concern [4].

Cyberchondria refers to the phenomenon of individuals experiencing escalated health anxiety and distress due to conducting online searches about medical symptoms, conditions, or perceived health risks [5]. With increasing reliance on the internet and online resources for health information, unfettered access to medical content could contribute to escalating anxiety and uncertainty in those predisposed to health worries [6]. The abundance of online health‐related material may trigger excessive reassurance‐seeking and fuel fears, especially when individuals have inadequate medical literacy to interpret the information found [7]. Cyberchondria shares traits such as the obsession of having a serious illness with somatic symptom disorder and sickness anxiety disorder [8].

The growing body of research highlights cyberchondria as a multifaceted issue influenced by psychological factors such as health anxiety, obsessive‐compulsive tendencies, and susceptibility to distress [9]. Studies indicate that it can lead to increased health worries, somatic symptoms, and depression, alongside practical consequences such as frequent, unnecessary healthcare visits and medical procedures [10, 11]. For example, research on university students has shown a prevalence rate of 37.5% among first‐year medical students in Indonesia, with higher rates observed among females [12, 13]. These results indicate that cyberchondria has a greater impact on specific population groups and has implications for both individuals and the healthcare system. Despite previous research on different aspects of cyberchondria, there is a lack of a comprehensive review of its frequency, demographic trends, psychological associations, and impacts on health. Comprehending these aspects is essential, as it forms the foundation for focused interventions and guides health policy on addressing the negative effects of excessive online health searches. This scoping review fills these gaps by looking at how common cyberchondria is, what factors are associated with it, how it varies among different demographics, what can predict it, and what its effects are in various populations and environments. The review helps enhance existing knowledge by summarizing current research, recognizing emerging trends, and pointing out gaps for future research. Moreover, it provides a basis for policymakers and healthcare professionals to create effective interventions to lessen the adverse effects of cyberchondria, decreasing unnecessary pressure on healthcare systems.

## 2. Methods

This scoping review was conducted according to the guidelines outlined by Arksey and O′Malley, which provide a systematic yet flexible approach suitable for mapping broad and heterogeneous evidence [14]. The steps recommended include (1) identifying and stating the research questions; (2) identifying relevant studies; (3) study selection; (4) data collection; (5) summary and synthesis of results; and (6) expert consultation. Unlike systematic reviews that focus on narrowly defined questions or intervention effectiveness, the Arksey and O′Malley framework allows for the inclusion of diverse study designs, enabling the identification of conceptual boundaries, evidence gaps, and research trends within this evolving field [15]. Moreover, previous scoping reviews addressing behavioral and digital health topics (e.g., [16, 17]) have successfully adopted this framework due to its adaptability and emphasis on summarizing complex evidence without imposing rigid quality assessment requirements.

The research questions for this scoping review include (1) What is the prevalence of cyberchondria? (2) What are the correlates of cyberchondria? (3) What are the demographic differences of cyberchondria? (4) What are the predictors of cyberchondria? (5) What are the effects of cyberchondria?

A comprehensive and systematic search strategy was developed to ensure broad and unbiased coverage of relevant literature on cyberchondria. Four major databases: PubMed, Scopus, JSTOR, and Dimensions, were purposefully selected for their multidisciplinary scope and extensive indexing of health, psychological, and behavioral research [18]. Medical Subject Headings (MeSH) terms were employed in PubMed to enhance search precision and ensure consistency in identifying studies on cyberchondria. These MeSH terms were subsequently refined and adapted for use in the other databases to account for variations in indexing and search functionalities [16].

The search strategy, including key terms, Boolean operators, and inclusion and exclusion criteria, is detailed in Table 1, whereas a sample PubMed search plan is presented in Table 2. The use of multiple databases and the adaptation of search terms across platforms aimed to reduce publication bias and enhance comprehensiveness, aligning with best practices for scoping reviews [19].

**Table 1:** Databases, inclusion, and exclusion criteria.

**Item**	**Search strategy**
Databases	PubMed, Scopus, JSTOR, Dimensions
Language	English
Time Filter	2000 or later
Spatial Filter	Global
Inclusion criteria	1. Peer‐reviewed literature 2. Published online from 2000 to later 3. Published in the English language 4. On the prevalence, correlates, demographic differences, predictors, and effects of cyberchondria. 5. Assessed by the Cyberchondria Severity Scale
Exclusion criteria	Reviews, abstracts, commentaries, letters to editors, and literature reviews.

**Table 2:** Search strategy in PubMed.

**Item**	**Search strategy**
# 1 Search to identify cyberchondria	Cyberchondria (MeSH term) OR excessive online health information seeking OR compulsive online health information seeking OR Internet health anxiety OR Internet self‐diagnosis.
#2 Search to identify prevalence	Prevalence (MeSH term) OR severity OR Incidence OR Extent OR Abundance OR Dominance OR commonness OR existence OR percentage.
# 3 Search to identify correlates	Correlates (MeSH term) OR factors OR relationships OR associates OR psychological factors OR personality factors OR behavioral factors.
#4 Search to identify demographic differences	Demographic differences (MeSH term) OR population differences OR socioeconomic differences OR sociodemographic differences OR group differences.
#5 Search to identify predictors	Predictors (MeSH term) OR indicators OR risk factors OR causal factors OR precursors.
#Search to identify effects	Effects (MeSH term) OR consequences OR outcomes OR impacts OR repercussions OR influence OR end results OR side effects
Overall search strategy	#2 AND #1 Not animal∗
	#3 AND #1 Not animal∗
	#4 AND #1 Not animal∗
	#5 AND #1 Not animal∗
Filters activated language:	English language Date: 1 January 2000

∗Is used to stress “Not animal” during the search to take out all studies that used animals as samples.

The searches were independently conducted by three reviewers (P.O., D.M., and a chartered librarian) to enhance the robustness and reproducibility of the search process. After each database search, regular meetings were held to compare results, discuss discrepancies, and reach consensus on the inclusion of records. This collaborative verification ensured that all potentially relevant studies were captured and that conflicts were resolved through discussion and mutual agreement. When necessary, a fourth opinion was sought from an external methodological advisor to validate decisions regarding inclusion or exclusion.

To ensure the comprehensiveness of the search, Mendeley reference management software was used to organize citations and automatically remove duplicates before screening. Google Scholar and Google were also searched to capture gray literature, in line with Arksey and O′Malley′s [14] recommendation to minimize publication bias. In addition, the reference lists of all eligible studies were manually screened to identify additional relevant records that may have been missed during database searching. The final search was completed on March 8, 2024.

Eligible studies were imported into Mendeley for data charting. Data were independently extracted by two reviewers (A.R. and B.A.M.E.) using predefined categories: author, year, country, study purpose, sample size, population, design, prevalence, correlates, and effects (see Table S1). To ensure inter‐rater reliability, discrepancies or conflicts between extractors were discussed in joint meetings and resolved through consensus. In instances where consensus could not be reached, a third reviewer (M.D.) adjudicated the disagreement.

Thematic analysis was conducted independently by three authors and resolved conflicts through regular meetings to draft the results of the study. Finally, to ensure the accuracy and completeness of the data and the relevance of the synthesized findings, an expert in cyberchondria research was consulted. This expert consultation, an optional but valuable stage recommended in the extended framework of Arksey and O′Malley [14], enhanced the validity, interpretability, and contextual richness of the results. All authors subsequently reviewed and approved the final dataset before thematic synthesis and reporting.

## 3. Results

### 3.1. Search Outcomes

**Figure 1:**
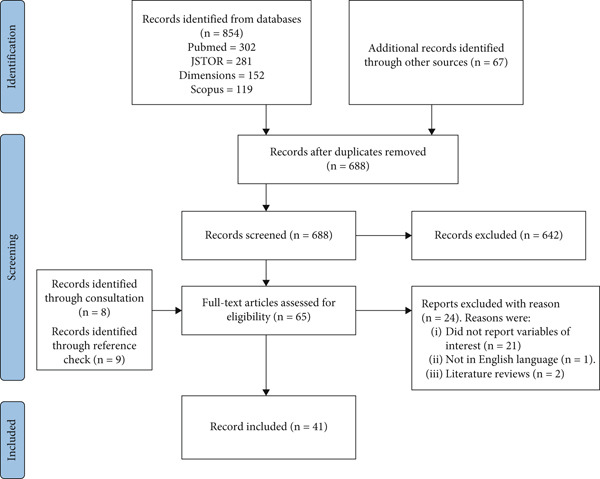
PRISMA flow chart.

The initial search across the four main databases (PubMed, Scopus, JSTOR, and Dimensions) yielded a total of 854 records. An additional 67 records were identified through Google Scholar and reference list checks. After removing duplicates using the Mendeley software, 688 records remained for further screening. Based on the eligibility criteria, 642 records were excluded during the title and abstract screening. Finally, 41 studies met the inclusion criteria and were included in this scoping review. The screening process is illustrated in the PRISMA Flow Chart (see Figure 1).

### 3.2. Study Characteristics

**Figure 2:**
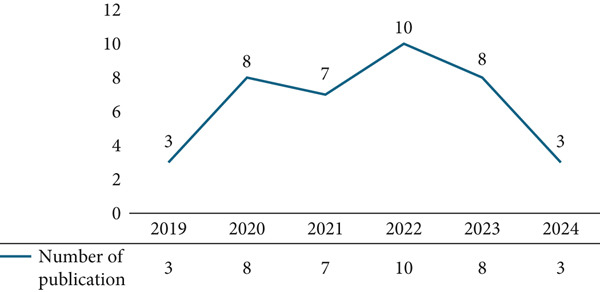
Year of publication.

**Figure 3:**
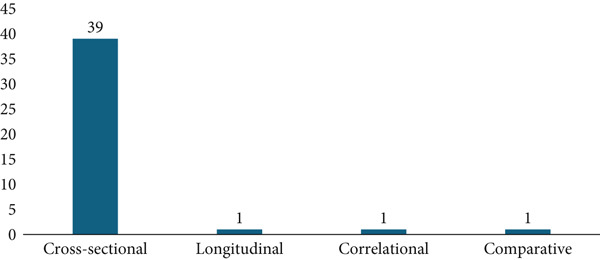
Research designs of included studies.

This scoping review identified 41 studies from 2015 to 2024 that met the inclusion criteria, providing data on the prevalence, correlates, demographic differences, predictors, and effects of cyberchondria. The studies were conducted in various countries, and the highest proportion was produced in Turkey (*n* = 8; 20.51%) (see Table 3). The highest number of articles synthesized in this review was published in 2022 (see Figure 2). Also, most of the studies utilized cross‐sectional survey designs (*n* = 35; 87.50%) (see Figure 3) with sample sizes ranging from 88 to 1615 participants.

**Table 3:** Distribution of articles by country.

**Country**	**Number of articles**	**Percentage**
Turkey	9	21.43%
India	5	11.90%
Pakistan	3	7.14%
United States of America	3	7.14%
Saudi Arabia	2	4.76%
Poland	2	4.76%
Egypt	1	2.38%
Brazil	1	2.38%
Croatia	3	7.14%
Italy	2	4.76%
Iran	1	2.38%
Jordan	1	2.38%
Nigeria	1	2.38%
Indonesia	1	2.38%
Germany	1	2.38%
China	1	2.38%
Bangladesh	1	2.38%
Australia, Ireland, Canada, New Zealand, United Kingdom and United States of America	1	2.38%
Europe French‐speaking countries	1	2.38%
Middle East and North Africa	1	2.38%

The populations studied included general adult population samples, university students, health professionals, and specific patient groups such as pregnant women and mothers of children with food allergies.

### 3.3. Prevalence of Cyberchondria

The results of this scoping review indicate that the prevalence of cyberchondria varies across different populations and geographical locations. The prevalence rate of cyberchondria ranged between 55.6% and 30.7%. The highest reported prevalence rate was 55.6% among information technology sector employees in India [20]. Another significant reported prevalence rate was 37.65% among first‐year medical students in Indonesia [12]. Similarly, the level of prevalence of cyberchondria was reported to range between lower and higher. A study reported a lower (14.2%) level of prevalence of cyberchondria among second‐year health students in Turkey [21]. In contrast, a study reported a higher level of prevalence among nursing students in Turkey [22]. In addition, El‐Zoghby et al. [23] found a moderate prevalence level of cyberchondria among medical students in Egypt (see Table 4).

**Table 4:** Prevalence of cyberchondria reported by included studies.

**Prevalence rate/levels**	**Country**	**Author/year**
55.6%	India	Makarla et al. (2019) [20]
42.5%	India	Pawar et al. (2022) [24]
37.65%	Indonesia	Aulia et al. (2020) [12]
37.5%	India	Kanganolli and Kumar (2020) [25]
30.9%	Poland	Ciułkowicz et al. (2022) [26]
30.7%	Canada, U.S. A	Mohammed et al. (2019) [27]
Higher	Turkey	Kan et al. (2023) [28]
Higher	Turkey	Varer Akpinar et al. (2023) [22]
Higher	Turkey	Uslu‐Sahan and Purtul (2023) [29]
Higher	Poland	Kobryn and Duplaga (2024) [30]
Higher	Bangladesh	Laato et al. (2020) [31]
Higher	Pakistan	Sabir and Naqvi (2023) [32]
Higher	Italy	Vismara et al. (2021) [33]
Higher	Iran	Wu et al. (2021) [34]
High	Saudi Arabia	Turkistani et al. (2020) [35]
High	Saudi Arabia	El‐Zayat et al. (2023) [36]
High	Turkey	Köse and Murat (2021) [37]
High	Brazil	Serra‐Negra et al. (2022) [38]
Moderate	Egypt	El‐Zoghby et al., (2024) [23]
Moderate to High	Nigeria	Abikoye and Lawal (2023) [39]
Moderate to High	Pakistan	Mubeen Akhtar (2019) [40]
Moderate	China	Peng et al. (2021) [41]
Moderate	Turkey	Uzun and Zencir (2022) [13]
Moderate	Turkey	Mrayyan, AL‐Atiyyat, et al. (2022) [42]
Moderate	Jordan	Mrayyan, Al‐Rawashdeh, et al. (2022) [43]
Lower	Turkey	Bati et al. (2018) [21]

### 3.4. Correlates of Cyberchondria

#### 3.4.1. Mental Health

Many mental health factors have been associated with cyberchondria all over the world. Some studies reported a positive association between internet addiction and cyberchondria severity [12, 22, 28, 38]. Also, intolerance of uncertainty has been identified to be positively correlated with cyberchondria [44]. Moreover, findings from other studies revealed that cyberchondria severity was positively correlated with health anxiety [26, 27, 39, 43, 45–48], Covid‐19 virus anxiety [49], and anxiety sensitivity [34]. Furthermore, a negative correlation has been found between general mental health and cyberchondria [20] (see Table 5).

**Table 5:** Correlates of cyberchondria.

**Main theme**	**Sub-theme**	**Author(s)**
Mental health	Anxiety	(Aulia et al., 2020 [12]; Kan et al. 2023 [28]; Serra‐Negra et al. 2022 [38]; Varer Akpinar et al. 2023 [22])
	Intolerance of uncertainty	(Batool 2022 [44]; Wu et al. 2021 [34])
	Health anxiety	(Abdelsattar et al. 2021 [45]; Abikoye and Lawal 2023 [39]; Ciułkowicz et al. 2022 [26]; Gioia and Boursier 2020 [46]; Infanti et al. 2023 [47]; Mohammed et al. 2019 [27]; Mrayyan, Al‐Rawashdeh, et al. 2022 [43]; Šoštarić et al. 2023 [48]; Vismara et al. 2021 [33])
	Covid‐19 virus anxiety	(Jungmann and Witthöft 2020 [49])
	Anxiety sensitivity	(Wu et al. 2021 [34])
	General mental health	(Makarla et al. 2019 [20])

Technological factors	Smartphone addiction	(El‐Zayat et al. 2023 [36])
	Internet addiction	(Mrayyan, Al‐Rawashdeh, et al. 2022 [43]; Mrayyan, AL‐Atiyyat, et al. 2022 [20]; Varer Akpinar et al. 2023 [22])
	Problematic internet use	(Vismara et al. 2021 [33])
	Internet literacy	(El‐Zayat et al. 2023 [36]; Köse and Murat 2021 [37])

#### 3.4.2. Technological Factors

Many technological factors have been shown to correlate with cyberchondria [13, 36]. For instance, a moderately positive correlation was determined between cyberchondria and smartphone addiction [37]. Also, a significant positive correlation was reported between cyberchondria and internet literacy [36] and internet addiction [22, 43]. Moreover, a positive correlation was found between cyberchondria and problematic usage of the internet [33] (see Table 5).

### 3.5. Demographic Differences of Cyberchondria

#### 3.5.1. Sociodemographic Factors

Gender differences were found in some studies; females had higher levels of cyberchondria compared with males [12, 27, 34, 38]. In contrast, other studies found that males engaged more in cyberchondria compared with females [25, 50]. Age differences were also reported; younger people, especially those below the age of 35, engaged more in cyberchondria [13, 29].

Cultural differences have also been demonstrated in cyberchondria; thus, a study reported that Brazilian dentists showed greater cyberchondria than Portuguese dentists [38]. Also, higher educational attainment, particularly having a master′s degree, was associated with increased cyberchondria engagement in one Chinese study [41] (see Table 6).

**Table 6:** Demographic differences in cyberchondria.

**Main theme**	**Sub-theme**	**Author(s)**
Sociodemographic factors	Gender	(Aulia et al. 2020 [12]; Kanganolli and Kumar 2020 [25]; Khazaal et al. 2021 [50]; Kobryn and Duplaga 2024 [30]; Mohammed et al. 2019 [27]; Serra‐Negra et al. 2022 [38]; Wu et al. 2021 [34])
	Age	(Khazaal et al. 2021 [50]; Kobryn and Duplaga 2024 [30]; Uzun and Zencir 2022 [13])
	Marital status	(Uslu‐Sahan and Purtul 2023 [29])
	Cultural	(Serra‐Negra et al. 2022 [38])
	Educational attainment	(Peng et al. 2021 [41])

### 3.6. Predictors of Cyberchondria

#### 3.6.1. Health‐Related Predictors

Presence of a health problem [21], negative medical experiences [27], child′s health problem [28], and self‐medication [10] were reported as predictors of cyberchondria. Also, prior frequent hospital visits [39] predicted cyberchondria (see Table 7).

**Table 7:** Predictors of cyberchondria.

**Main theme**	**Sub-theme**	**Author(s)**
Health‐related predictors	Presence of a health problem	(Bati et al. 2018 [21])
	Negative medical history	(Mohammed et al. 2019 [27])
	Child′s health problem	(Kan et al. 2023 [28])
	Prior frequent hospital visits	(Abikoye and Lawal 2023 [39])
	Self‐medication	(Khan and Pandey 2022 [10])

Psychological predictors	General anxiety	(Gioia and Boursier 2020 [46])
	Metacognitive beliefs	(Fergus 2013 [51])
	Higher levels of health anxiety	(Peng et al. 2021 [41])
	Increased health anxiety	(Turhan Cakir 2022 [52])
	Anxiety sensitivity	(Batool 2022 [44]; Norr et al. 2015 [53])
	Trait health anxiety	(Jungmann and Witthöft 2020 [49])
	Health anxiety	(Jokic‐Begic et al. 2020 [54]; Kobryn and Duplaga 2024 [30])
	Depression	(Gioia and Boursier 2020 [46])
	Stress	(Gioia and Boursier 2020 [46])
	Fear and anxiety	(Wu et al. 2021 [34])
	Trait impulsivity	(Eşkisu et al. 2024 [55])

Covid‐19 related factors	Professional inactivity	(Ciułkowicz et al. 2022 [26])
	Limited access to healthcare	(Ciułkowicz et al. 2022 [26])
	Covid‐19 symptoms	(Abdelsattar et al. 2021 [45]).
	Fear of contracting covid‐19 virus	(Infanti et al. 2023 [47])

Technological factors	Compulsive internet use	(Kanganolli and Kumar 2020 [25]; Khazaal et al. 2021 [50])

#### 3.6.2. Psychological Predictors

Table 7 presents predictors of psychological predictors of cyberchondria. The table shows that some significant predictors of cyberchondria included health anxiety [30, 54]. Also, trait health anxiety emerged as a significant predictor of cyberchondria [49]. Increased health anxiety predicted cyberchondria among women with high‐risk HPV [52]. Furthermore, general anxiety, depression, and stress [46], fear and anxiety [34], and trait impulsivity [55] were reported as predictors of cyberchondria. Another potential risk factor was anxiety sensitivity [53]. Additionally, cyberchondria was found to share moderate to strong associations with metacognitive beliefs [56].

#### 3.6.3. COVID‐19 Pandemic Factors

A study reported professional inactivity and subjectively limited access to healthcare due to COVID‐19 to be predictors of cyberchondria [26]. In addition, fear of contracting the COVID‐19 virus [47] and experiencing COVID‐19 symptoms [45] were identified as predictors of cyberchondria (see Table 7).

#### 3.6.4. Technological Factors

Kanganolli and Kumar [25] reported that access to the internet and duration of internet use predicted cyberchondria among students (see Table 7).

### 3.7. Effects of Cyberchondria

#### 3.7.1. Health Effects

Health effects of cyberchondria reported include self‐diagnosis [32] and self‐medication [10] (see Table 8).

**Table 8:** Effects of cyberchondria.

**Main theme**	**Sub-theme**	**Author(s)**
Health effects	Self‐diagnosis	(Sabir and Naqvi 2023 [32])
	Self‐medication	(Uzun and Zencir 2022 [13])
	Heightened threat perception	(Kanganolli and Kumar 2020 [25])

Behavioral effects	Increased safety behaviors	(Jokic‐Begic et al. 2020 [54])
	Avoidance of social situations	(Ciułkowicz et al. 2022 [26])
	Troubles with sleep	(Mubeen Akhtar 2019 [40])

Academic effects	Lower academic performance	(Patanapu et al. 2022 [57])

Psychological effects	Reduced trust in physicians	(Khan and Pandey 2022 [10])
	Health anxiety	(Kanganolli and Kumar 2020 [25]; Turkistani et al. 2020 [35]).
	Distress	(Mubeen Akhtar 2019 [40]; Šoštarić et al. 2023 [48])
	Compulsive search	(Šoštarić et al. 2023 [48])
	Heightened threat perception	(Kanganolli and Kumar 2020 [25])

#### 3.7.2. Behavioral Effects

The behavioral effects of cyberchondria included increased safety behaviors during the COVID‐19 pandemic [54], troubles with sleep [40], and avoidance of social situations [26] (see Table 8).

#### 3.7.3. Academic Effects

Cyberchondria affected the academic performance of undergraduate dental students, resulting in lower academic performance [57] (see Table 8).

#### 3.7.4. Psychological Effects

The psychological effects of cyberchondria included increased distress and compulsive searching among pregnant women [48], increased anxiety and distress among graduates [40], increased health anxiety [35], and heightened threat perception [25]. Other psychological effects found were reduced trust in physicians [10] and troubles with sleep [40] (see Table 8).

## 4. Discussion

This scoping review provides a comprehensive overview of the prevalence, correlates, demographic differences, predictors, and effects of cyberchondria. The prevalence of cyberchondria varied across different populations and geographical locations, with rates ranging from 30.7% to 55.6%. Psychological factors such as health anxiety, anxiety sensitivity, and intolerance of uncertainty were identified as correlates and predictors of cyberchondria. Demographic differences were observed in gender, age, parental status, culture, and education level. The effects of cyberchondria encompass heightened health anxiety, safety behaviors, social avoidance, self‐diagnosis, self‐medication, and reduced trust in physicians.

### 4.1. Prevalence of Cyberchondria

The prevalence rate of cyberchondria varied widely across the included studies, ranging from as low as 30.7% in Canada and the United States [27] to as high as 55.6% in India [20]. Higher prevalence levels were reported in several studies in Turkey—among medical students [23], nursing students [22], social media users [29], and mothers of children with food allergies [28]. In contrast, a study reported a lower prevalence level among health students in Turkey [21]. This inconsistency in prevalence rates and levels may be attributed to differences in study populations, geographical locations, and assessment methods. The highest prevalence rate was reported among information technology sector employees in India [20], which could be due to their increased exposure to technology and the Internet. Medical students and healthcare professionals also showed higher prevalence rates [12, 25, 38], possibly due to their heightened awareness of health‐related information and the potential for self‐diagnosis. The variability in prevalence rates and levels may be attributed to differences in healthcare systems, internet access, and cultural factors. These findings emphasize the need for tailored interventions and public health strategies to address cyberchondria, acknowledging the diverse landscape of online health information‐seeking behaviors worldwide. This review highlights the importance of continued research and monitoring of cyberchondria prevalence to inform effective healthcare responses and promote healthy online behaviors.

### 4.2. Correlates of Cyberchondria

Mental health factors, such as internet addiction [12, 28], intolerance of uncertainty [44], health anxiety [39, 45, 47], Covid‐19 virus anxiety [49], and anxiety sensitivity [34], all demonstrate a positive correlation with cyberchondria. Intolerance of uncertainty shares a distinctive link with excessiveness. The reason is that people with an elevated level of intolerance of uncertainty have developed an uncontrollable desire to overcome the uncertainty of events, whether they are future‐related (inhibitive intolerance of uncertainty) or present‐related (prospective intolerance of uncertainty); that is why they use digital mediums for online medical information to reduce the anxiety associated with the loss of controllability [53]. This suggests that individuals struggling with these mental health concerns may be more likely to engage in excessive online health searching, which is commonly referred to as cyberchondria.

Conversely, technological factors like smartphone addiction [37], internet literacy [36], internet addiction [43], and problematic internet usage [33] also show a positive correlation with cyberchondria. This indicates that individuals with higher technological engagement and literacy may be more susceptible to cyberchondria. Thus, the more time you spend with your smartphone online, the more likely you are to engage in cyberchondria. In other words, the association between cyberchondria and problematic internet use, including internet addiction, suggests that compulsive health‐related searches may be part of a broader pattern of maladaptive internet behaviors [33, 42, 43, 56]. This finding highlights the need for interventions that address both cyberchondria and problematic internet use to promote healthy online behaviors.

The sole exception is general mental health [20], which exhibited a negative correlation with cyberchondria, implying that individuals with poor mental health may be more likely to engage in cyberchondria.

### 4.3. Demographic Differences in Cyberchondria

Gender differences in cyberchondria were inconsistent across studies. While some studies reported higher levels of cyberchondria among females [12, 27, 34, 38], others found that males were more likely to engage in cyberchondria [25, 50]. Female students were more likely to search for online information after noticing unexplained symptoms in their bodies, to consult doctors or other medical specialists after finding troubling online information, and to openly discuss the results of online research with medical professionals [12]. These discrepancies may be due to variations in sample characteristics and cultural factors. Further research is needed to clarify the role of gender in cyberchondria and to explore potential moderating variables.

Age differences in cyberchondria were more consistent, with younger individuals, particularly those under 35, showing higher levels of cyberchondria [13, 29]. This finding may be attributed to younger adults′ greater familiarity with technology and the internet, as well as their increased exposure to health‐related information online. Interventions targeting younger populations may be particularly effective in preventing and reducing cyberchondria.

Parental status, cultural differences, and education level also emerged as demographic factors associated with cyberchondria. Owing to the developing technology, increasing internet usage, and the widespread use of internet communication, a growing number of mothers of children with food allergies have easy access to health‐related information and thus engage in cyberchondria [28]. A notable cultural difference in cyberchondria was identified between dentists in Brazil and Portugal, with Brazil having a higher number of cyberchondriacs despite similar proportions of internet users in both countries (70%). The implications of this finding are significant, suggesting that cultural tailoring of interventions may be necessary to effectively address cyberchondria in different cultural contexts. Further research is needed to explore the specific cultural factors at play and to develop culturally sensitive interventions that promote healthy online behaviors. These findings suggest that specific life circumstances and health literacy may influence individuals′ propensity to seek out health information online. Tailored interventions addressing the unique needs and concerns of these groups may be beneficial in reducing cyberchondria.

### 4.4. Predictors of Cyberchondria

Health anxiety emerged as a significant predictor of cyberchondria across multiple studies [27, 28, 39, 45–47, 49]. Individuals with heightened health anxiety may be more prone to engaging in excessive online health information seeking to alleviate their concerns. This finding aligns with the cognitive‐behavioral model of health anxiety, which suggests that health‐related fears and beliefs can drive maladaptive coping behaviors, such as reassurance seeking through internet searches [58].

Anxiety sensitivity and intolerance of uncertainty were also identified as predictors of cyberchondria [34, 44, 46, 48, 53]. The two potential risk factors (intolerance of uncertainty and anxiety sensitivity) share many common features and are positively linked to reassurance‐seeking and excessiveness, and the elevated levels of these two variables may lead to cyberchondria [59]. In essence, individuals with high anxiety sensitivity may be more likely to misinterpret bodily sensations as signs of serious illness, leading to increased health‐related fears and cyberchondria. Similarly, those with low tolerance for uncertainty may engage in cyberchondria to seek out health information online to reduce ambiguity and gain a sense of control over their health [51].

### 4.5. Effects of Cyberchondria

The included studies reported various negative effects of cyberchondria on individuals′ well‐being and healthcare utilization. Heightened health anxiety and distress were commonly reported consequences [25, 35], which may perpetuate a vicious cycle of increased online health information seeking and escalating anxiety. Cyberchondria was also linked to increased safety behaviors [54] and avoidance of social situations [26], indicating a broader impact on individuals′ daily functioning and quality of life. These effects highlight the importance of addressing cyberchondria as a public health concern. Interventions aimed at mitigating cyberchondria should consider the cognitive, emotional, and behavioral aspects of this phenomenon to effectively reduce its impact on individuals′ daily lives.

Self‐diagnosis and self‐medication behaviors were associated with cyberchondria in some studies [10, 32], highlighting the potential risks of relying on online health information without professional guidance. These behaviors can lead to delayed or inappropriate treatment, as well as adverse health outcomes. Healthcare providers should be aware of the potential for cyberchondria among their patients and provide accurate, reliable health information to counteract the negative effects of online searches.

Cyberchondria was also found to reduce trust in physicians [10], which can have implications for the patient–provider relationship and adherence to medical advice. Healthcare providers may need to address patients′ concerns stemming from online health information and provide reassurance to maintain trust and effective communication.

The negative impact of cyberchondria on academic performance [57] shows the importance of addressing this issue among student populations. Educational institutions should consider implementing awareness programs and support services to help students manage health‐related anxieties and promote healthy online behaviors.

### 4.6. Limitations

This review might be influenced by publication bias due to its emphasis on published literature, which could lead to the exclusion of potentially valuable unpublished studies. Moreover, the review′s inclusivity may be limited by the decision to include only English‐language articles, potentially overlooking important research published in other languages. Lastly, the comprehensiveness of the review might be affected by the specificity of the eligibility criteria and the restrictions placed on keywords, which could have resulted in the omission of relevant studies.

Another potential limitation is that the included studies primarily used cross‐sectional designs, which limit the ability to establish causal relationships between cyberchondria and its correlates, predictors, and effects. The included studies also relied on self‐report measures of cyberchondria, which may be subject to response bias and social desirability. Third, the included studies were conducted in various countries and cultural contexts, which may limit the generalizability of the findings.

### 4.7. Implications for Policy and Practice

The findings of this scoping review have important implications for healthcare providers, mental health professionals, and public health initiatives. Healthcare providers should be aware of the potential for cyberchondria among their patients and take steps to address health‐related anxieties and provide accurate, reliable health information. This may involve discussing patients′ online health information‐seeking behaviors during consultations, providing guidance on reputable health information sources, and offering reassurance to alleviate anxiety. Mental health professionals can play a crucial role in identifying and treating individuals with cyberchondria. Cognitive‐behavioral interventions targeting health anxiety, anxiety sensitivity, and intolerance of uncertainty may be effective in reducing cyberchondria and its associated distress. Mental health professionals should also be aware of the potential co‐occurrence of cyberchondria and problematic internet use and address these issues concurrently. Public health initiatives should focus on promoting digital health literacy and responsible online health information‐seeking behaviors. This may involve developing and disseminating educational materials on evaluating the credibility of online health information, encouraging individuals to consult with healthcare professionals before making health‐related decisions based on online searches, and raising awareness about the potential negative consequences of cyberchondria.

### 4.8. Recommendations for Future Studies

Future studies should aim to address the gaps identified in this review by establishing a standardized definition of cyberchondria, using validated measurement tools, and conducting longitudinal studies to provide valuable insights into the development, progression, and long‐term effects of cyberchondria on mental health, healthcare utilization, and quality of life. Additionally, there is a need for more research involving diverse populations, considering factors such as age, gender, cultural background, and socioeconomic status, to better understand the demographic differences in cyberchondria prevalence and correlates, as well as investigating potential mediating and moderating factors, such as health anxiety, intolerance of uncertainty, and eHealth literacy, to identify risk and protective factors. Furthermore, future studies should compare cyberchondria with offline health anxiety to distinguish the unique features and effects of excessive online health‐related searches, examine the impact of cyberchondria on healthcare systems, and develop and evaluate targeted interventions, such as cognitive‐behavioral therapy or psychoeducation, to mitigate its negative effects and improve overall well‐being. Qualitative research, such as interviews or focus groups, could provide a deeper understanding of the subjective experiences and motivations of individuals with cyberchondria, while investigating the role of technology, such as search algorithms, personalization, and social media, could inform potential strategies for prevention and management. Lastly, encouraging collaboration among researchers, healthcare professionals, and technology experts, as well as promoting open science practices, could accelerate progress in understanding and addressing cyberchondria.

## 5. Conclusion

The pervasive issue of cyberchondria encompasses a complex interplay of psychological, social, and technological factors. These multifaceted risk factors, rooted in health anxiety, intolerance of uncertainty, and the abundance of online health information, significantly impact the mental well‐being and healthcare‐seeking behaviors of individuals. The implications of cyberchondria span from immediate distress and anxiety to long‐term consequences affecting mental health, healthcare utilization, and quality of life. Cyberchondria′s profound impact extends beyond the individual, potentially straining healthcare systems and perpetuating cycles of anxiety and misinformation. Addressing these challenges demands comprehensive measures, advocating for digital health literacy, evidence‐based online health resources, and the integration of cyberchondria awareness into healthcare practices. Tailored interventions, such as cognitive‐behavioral therapy and psychoeducation, along with collaborative efforts across healthcare, research, and technology sectors, are imperative to mitigate cyberchondria′s detrimental effects on individuals and society. While this scoping review offers valuable insights, future studies should focus on longitudinal research, diverse populations, mediating and moderating factors, comparisons with offline health anxiety, and the impact on healthcare systems to deepen our understanding and enhance effective strategies to address cyberchondria in the digital age.

## Ethics Statement

The authors have nothing to report.

## Consent

The authors have nothing to report.

## Disclosure

All authors read and approved the final manuscript.

## Conflicts of Interest

The authors declare no conflicts of interest.

## Author Contributions

Dr. Daniel Miezah and Paul Obeng conceptualized the study and conducted the literature search. Rejoice Adzakpa and Emmanuella Mawuena Ama Bani conducted the data extraction. Daniel Miezah and Paul Obeng wrote the methodology. Rejoice Adzakpa and Emmanuella Mawuena Ama Bani conducted the formal analysis and drafted the manuscript. Daniel Miezah and Paul Obeng reviewed the manuscript. Supervision: Dr. Daniel Miezah.

## Funding

No funding was received for this manuscript.

## Supporting information


**Supporting Information 1.** Additional supporting information can be found online in the Supporting Information section. Table S1: Data extraction.

## Data Availability

Data are available in the article′s supplementary material.
