# Supplementary material for: Prevalence and Associated Factors of Cyberchondria: A Scoping Review
Source: ScientificWorldJournal. 2026 Jan 22;2026:9950027. doi: 10.1155/tswj/9950027 (PMC12825022; doi:10.1155/tswj/9950027)
Supplement: Supplementary file 1 — Supporting Information 1. Additional supporting information can be found online in the Supporting Information section. Table S1: Data extraction. [file TSWJ-2026-9950027-s001.doc]

Table 9. Data Extraction

| Author(s), year of publication, and country of origin | Purpose of the study | Study design | Population | Sample size | Prevalence of cyberchondria | Correlates of cyberchondria | Demographic differences of cyberchondria | Predictors of cyberchondria | Effects of cyberchondria |
| --- | --- | --- | --- | --- | --- | --- | --- | --- | --- |
| Aulia et al. (2020)  Indonesia | To measure the level of cyberchondria severity of first-year medical students | Cross-sectional | First-year medical students of Yogyakarta | 162 students | 37.65% | Anxiety | Gender |  |  |
| Uzun and Zencir (2022)  Turkey | To determine the level of cyberchondria and related factors in university employees | Cross-sectional | Academic and non-academic university staff | 335 | Moderate |  | Age |  | Self-medication |
| Kan et al. (2023)  Turkey | to evaluate the relationship between cyberchondria and socioeconomic factors | Cross-sectional | Mothers of children with food allergies and mothers of healthy children | 120 | Higher | Educational level and anxiety | Physical illness | Child’s health problems |  |
| Sabir and Naqvi (2023)  Pakistan | investigate the prevalence of cyberchondria and self-diagnosis behaviour among university students | Cross-sectional | University students | 500 | Higher |  |  |  | Self-diagnosis |
| Turkistani et al. (2020)  Saudi Arabia | to investigate the prevalence of cyberchondria and the impact of social media among university students | Cross-sectional | University students | 651 | High |  |  |  | Increased health anxiety |
| Mubeen Akhtar (2019)  Pakistan | To study the cyberchondria and related worries about health among individuals without any diagnosed medical condition. | Cross-sectional | Graduates | 150 | Moderate to high |  |  |  | Sleep troubles |
| Serra-Negra et al. (2022)  Brazil | To assess cyberchondria level and associated factors | Cross-sectional | Brazilian and Portuguese dentists | 597 | High | Anxiety | Gender, Occupational differences |  |  |
| Köse and Murat (2021)  Turkey | To examine the relationship between adolescents' smartphone addiction and cyberchondria | Correlational | High school students | 395 | High | Internet use |  |  |  |
| Abikoye and Lawal (2023)  Nigeria | To investigate the prevalence and predictors of cyberchondria among Nigerians during the COVID-19 pandemic | Cross-sectional | Literate Nigerians | 406 | Moderate to high. | Health anxiety |  | Number of prior hospital visits |  |
| Makarla et al. (2019)  India | To assess the prevalence and factors influencing cyberchondria among employees working in the information technology sector | Cross-sectional | Information technology sector employees | 205 | 55.6% | poor mental health |  |  |  |
| Vismara et al. (2021)  Italy | To investigate behavioral patterns in online health-related searches and Cyberchondria during the COVID-19 pandemic time | Cross-sectional | Italian general population | 572 | Higher | Health anxiety, problematic internet usage |  |  |  |
| Ciułkowicz et al. (2022)  Poland | To estimate the level of cyberchondria and describe the socio-demographic, clinical and pandemic-related factors | Cross-sectional | Adult Polish Population of Internet users | 538 | 30.9% | Health anxiety |  | Professional inactivity and limited access to healthcare | Less eager to engage in social meetings |
| Varer Akpinar et al. (2023)  Turkey | To determine the relationship between cyberchondria and COVID-19 anxiety and internet addiction | Cross-sectional | Nursing students | 843 | Higher | Internet addiction and anxiety |  |  |  |
| El-Zayat et al. (2023)  Saudi Arabia | To assess the prevalence of cyberchondria and its association with smartphone addiction | Cross-sectional | Adult Saudis living in Jeddah | 418 | High | Smartphone addiction and high internet literacy |  |  |  |
| Šoštarić et al. (2023)  Croatia | To explore the risk factors, triggers, and outcomes of cyberchondria in pregnant women | Longitudinal | Pregnant women | 149 |  | Health anxiety and anxiety sensitivity |  |  | Increased distress and compulsive search |
| Infanti et al. (2023)  Europe French-speaking countries | To investigate changes in the severity of cyberchondria during the COVID-19 pandemic and identify the predictors of cyberchondria | Cross-sectional | Residents fluent in French | 725 |  | Health anxiety |  | Covid-19 related fears |  |
| Jungmann and Witthöft (2020)  Germany | To examine the associations between trait health anxiety, cyberchondria, coping strategies and anxiety related to Covid-19 | Cross-sectional | German general population | 1615 |  | Current virus anxiety |  | Trait health anxiety |  |
| Jokic-Begic et al. (2020)  Croatia | To examine how cyberchondria is related to changes in levels of Covid-19 concern and safety behaviours | Cross-sectional | Croatian general population | 888 |  |  | Stronger in the first wave | Health anxiety | Increased safety behaviours |
| Jokic-Begic et al. (2020)  Croatia | To examine how cyberchondria is related to changes in levels of Covid-19 concern and safety behaviours | Cross-sectional | Croatian general population | 966 |  |  | Stronger in first wave of Covid-19 | Health anxiety | Increased safety behaviours |
| Bati et al. (2018)  Turkey | To determine the factors affecting cyberchondria and health anxiety levels in health sciences students | Cross-sectional | 2^nd^ year students (medicine, pharmacy, nursing and dentistry) | 874 | Lower |  |  | Presence of a health problem |  |
| Gioia and Boursier (2020)  Italy | To test the predictive role of perceived feelings of loneliness, general anxiety, depression and stress on cyberchondria | Cross-sectional | Italian women | 247 |  | High anxiety |  | General anxiety, loneliness, depression and stress |  |
| Wu et al. (2021)  Iran | To investigate the associations between fear of COVID-19, COVID-19 anxiety, and cyberchondria | Cross-sectional | Residents fluent in Persian language | 694 | Higher | Intolerance of uncertainty and anxiety sensitivity | Gender | Fear and anxiety |  |
| Mrayyan, AL‐Atiyyat, et al. (2022)  Turkey | To investigate students cyberchondria and addiction levels and to examine whether cyberchondria predicts internet addiction | Cross-sectional | University students | 143 | Moderate | Internet addiction |  |  |  |
| (Mrayyan, Al-Rawashdeh, et al., 2022)  Jordan | To compare university-level students’ scores in cyberchondria, IA, and anxiety (i.e. anxiety sensitivity, health anxiety, and coronavirus anxiety) based on students’ characteristics of number of years online, grade point average (GPA), the field of study, year of study, gender, and the availability of Internet access at school | Comparative study | University students | 143 | moderate | Internet addiction, anxiety sensitivity and health anxiety | Low grades |  |  |
| (Norr et al., 2015)  U.S.A | To investigate Anxiety Sensitivity and Intolerance Uncertainty as potential risk factors for cyberchondria while controlling for health anxiety | Cross-sectional | Community sample recruited through Amazon's Mechanical Turk | 526 |  |  |  | Anxiety sensitivity |  |
| (Mohammed et al., 2019)  Canada and U.S.A | To  investigates how the development of cyberchondria relates to characteristics of the person searching the internet | Cross-sectional | Author’s own social network and social media | 191 | 30.7% | Health anxiety | Gender | Negative medical history |  |
| Peng et al. (2021)  China | To investigate the status and influencing factors of cyberchondria in residents in China during the epidemic period of COVID-19 | Cross-sectional | Community residents of Nanyang city | 674 | Moderate |  | Higher educational level | Higher levels of health anxiety |  |
| Khazaal et al. (2021)  Australia, Ireland, Canada, New Zealand, U.K & U.S.A | To assess compulsive health-related Internet use in relation to cyberchondria while controlling for related variables | Cross-sectional | Adult participants from an online platform | 749 |  |  | Gender | Compulsive internet use |  |
| Laato et al. (2020)  Bangladesh | To test why people share unverified COVID-19 information through social media. | Cross-sectional | Social media users | 294 | Higher |  |  | Age |  |
| Sahan and Purtul (2023)  Turkey | To investigate whether women’s cyberchondria levels differ according to some sociodemographic demographic characteristics and the effect of health anxiety and e-health literacy on cyberchondria levels. | Cross-sectional | Gynecology outpatient in a state hospital | 178 | Higher |  | Age and marital status |  |  |
| Abdelsattar et al. (2021)  Middle East & North Africa | To investigate the relationship among Cyberchondria severity level, health anxiety and health locus of control as a direct response for COVID-19 anxiety among Arab people in the Middle East & North Africa (MENA) and the Gulf States | Cross-sectional | Arabs in Middle East and Gulf states | 573 |  | Health anxiety |  | Covid-19 symptoms |  |
| Batool (2022)  Pakistan | To investigate the relationship between intolerance of uncertainty and cyberchondria and to find out the mediating role of anxiety sensitivity in this relationship | Cross-sectional | Southern Punjab residents aged 18 to 55 years | 413 |  | Intolerance of uncertainty |  | Anxiety sensitivity |  |
| Kanganolli and Kumar (2020)  India | To estimate the prevalence of cyberchondria and assess factors influencing cyberchondria among university students | Cross-sectional | Undergraduate medical students | 136 | 37.5% |  | Gender | Internet use | Heightened threat perception and health anxiety |
| Khan and Pandey (2022)  India | To investigate the negative behavioral consequences of cyberchondria that pose health risks to users. | Cross-sectional | General population | 317 |  |  |  | Self-medication | Reduces patient’s trust in physicians |
| Pawar et al. (2022)  India | To assess the prevalence of cyberchondria and its constructs among patients with metabolic syndrome in India | Cross-sectional | Tertiary care hospital | 379 | Moderate to severe |  |  |  |  |
| Fergus and Spada (2017)  U.S.A | To provide the first known direct examination of an association between cyberchondria and Problematic Internet Use | Cross-sectional | U.S community adults | 597 |  |  |  | Metacognitive beliefs |  |
| Turhan Cakir (2022)  Turkey | To investigate the level of cyberchondria in patients with high-risk HPV positivity. | Cross-sectional | Women diagnosed with high-risk Human Papillomavirus (HPV) | 140 |  |  |  | Increased health anxiety |  |
| Patanapu et al. (2022)  India | To investigate the prevalence of Cyberchondria and its impact on the academic performance of undergraduate dental students | Cross-sectional | Undergraduate dental students | 302 |  |  |  |  | Lower academic performance |
| Kobryn and Duplaga (2024)  Poland | To examine the prevalence of cyberchondria among adult internet users | Cross-sectional | Adult internet users | 1613 | Higher |  | Age, gender | Health anxiety |  |
| El-Zoghby et al. (2024)  Egypt | To explore the rising phenomenon of smartphone addiction and its potential role in cyberchondria | Cross-sectional | Medical students | 1435 | Moderate |  |  |  |  |
| Eşkisu et al. (2024)  Turkey | To converge a structural equation model to unfold the compositive relationships between trait impulsivity, health cognitions, metacognitions about health, fear of COVID-19, and cyberchondria | Cross-sectional | Online | 651 |  |  |  | Trait impulsivity |  |
